# The Human Milk Microbiota is Modulated by Maternal Diet

**DOI:** 10.3390/microorganisms7110502

**Published:** 2019-10-29

**Authors:** Marina Padilha, Niels Banhos Danneskiold-Samsøe, Asker Brejnrod, Christian Hoffmann, Vanessa Pereira Cabral, Julia de Melo Iaucci, Cristiane Hermes Sales, Regina Mara Fisberg, Ramon Vitor Cortez, Susanne Brix, Carla Romano Taddei, Karsten Kristiansen, Susana Marta Isay Saad

**Affiliations:** 1 School of Pharmaceutical Sciences, University of São Paulo, São Paulo 05508-000, SP, Brazil; 2 Food Research Center (FoRC), University of São Paulo, São Paulo 05508-000, SP, Brazil; 3 Laboratory of Genomics and Molecular Biomedicine, Department of Biology, University of Copenhagen, DK-2100 Copenhagen, Denmark; 4 School of Public Health, University of São Paulo, São Paulo 01246-904, SP, Brazil; 5 Department of Biotechnology and Biomedicine, Technical University of Denmark, DK-2800 Kgs. Lyngby, Denmark; 6 School of Arts, Sciences and Humanities, University of São Paulo, São Paulo 03828-000, SP, Brazil

**Keywords:** maternal diet, microbiota, breast milk, gut colonization, breastfeeding

## Abstract

Human milk microorganisms contribute not only to the healthy development of the immune system in infants, but also in shaping the gut microbiota. We evaluated the effect of the maternal diet during pregnancy and during the first month of lactation on the human milk microbiota in a cross-sectional study including 94 healthy lactating women. Microbiota composition was determined by 16S rDNA profiling and nutrient intake assessed through food questionnaires. Thirteen genera were present in at least 90% of all samples, with three genera present in all samples: *Streptococcus*, *Staphylococcus*, and *Corynebacterium*. Cluster analysis indicated two distinct compositions: one marked by a high abundance of *Streptococcus* (cluster 1), and other by a high abundance of *Staphylococcus* (cluster 2). A global association with milk microbiota diversity was observed for vitamin C intake during pregnancy (*p* = 0.029), which was higher for cluster 2 individuals (cluster 2 median = 232 mg/d; cluster 1 = 175 mg/d; *p* = 0.02). Positive correlations were found between *Bifidobacterium* in the milk and intake of polyunsaturated and linoleic fatty acids during the lactation period (*p* < 0.01). We show that maternal diet influences the human milk microbiota, especially during pregnancy, which may contribute in shaping the gut microbiota.

## 1. Introduction

Breast-fed babies develop distinct gut microbiota patterns, more enriched in *Bifidobacterium* and *Lactobacillus* compared to formula-fed babies, which are enriched in species belonging to *Clostridia* [1]. Such influence is thought to contribute the correct development of the immune system, and improved health outcomes associated with breastfeeding [2].

There are very few studies on how or even which microorganisms present in human milk assist on colonizing the infant gastrointestinal tract, as well as their potential role in the mother and their infant’s health [3,4]. Species already isolated from human milk include *Lactobacillus gasseri*, *L. rhamnosus*, *L. plantarum*, *L. fermentum*, *Enterococcus faecium*, *Bifidobacterium breve*, *B. adolescentis*, *B. bifidum*, *B. longum*, and *B. dentium* [5,6,7]. However, studies employing high throughput sequencing have shown that human milk contains a larger diversity of bacterial species comprising more than 207 bacterial genera [4]. Among these genera, *Staphylococcus* and *Streptococcus* are the predominant bacterial genera [4,8], and it is thought that 12 core genera account for as much as 81% of all bacteria present in human milk after the first month post-delivery [4]. Nevertheless, there is also a large difference in diversity among samples, and it remains unclear what such high inter-personal variation may mean for the newborn microbiome [9].

Pre-gestational body mass index (BMI) [9,10], lactation time [10], mode of delivery [10,11] or even the geographic location [11] have all been related to the differences in human milk microbiota composition. In addition, maternal diet [12] and nutrient content in human milk [11,13] have been recently proposed as factors that may influence its microbiota composition.

How the milk microbiota is modulated is a matter of debate One hypothesis is that the mother’s diet may help in shaping the human milk microbiota. This is substantiated by previous studies reporting that specific nutrients from the maternal intake affect the nutrient composition of the human milk [14,15]. An alternative hypothesis proposes an entero-mammary pathway as a route for bacteria to reach the human milk, enabling the transfer of bacteria from the maternal gastrointestinal tract to the mammary gland [13,16]. The diet influences the gut microbiota [17,18] also during the perinatal period [19], the latter hypothesis suggests that the diet might influence the human milk microbiota diversity via changes in the maternal gut microbiota diversity. This might explain the origin of bacteria in milk that are not found on the maternal skin or in the infant’s mouth, which are described as potential bacterial sources shaping the human milk microbiota [16,20,21].

Since vertical transfer of bacteria via breastfeeding has been suggested as an important contributor to the initial establishment of the microbiota in the developing infant gut [4], it is important to understand factors that might influence the composition of the human milk microbiota, especially the role of maternal diet for which limited information is available. To our knowledge, the relation between the maternal diet during pregnancy and the milk microbiota has not yet been explored. Here we evaluated the effect of the maternal diet during pregnancy and during the first month of the lactation period on the human milk microbiota profile.

## 2. Materials and Methods

### 2.1. Subjects and Study Design

A cross-sectional investigation was carried out including healthy lactating women volunteers of 18 to 37 years old with uncomplicated pregnancy. Women who had vaginal deliveries at the University Hospital, University of São Paulo, São Paulo city, between September 2014 and June 2016 were assessed for eligibility for inclusion into the study. Inclusion criteria were having babies born between week 37 and 42 of pregnancy with weight between the 10th and 90th percentiles (adequate weight for gestational age), according to the United States reference curve for fetal growth [22], having normal bowel frequency (minimum once every 2 days, maximum 3 times per day), and breastfeeding. The volunteers were screened through medical records and from oral information. Exclusion criteria were current chronic gastrointestinal disease, genetic disease, cardiac disease, kidney disease, hypertension, diabetes mellitus, immunodeficiency diseases, eclampsia, gestational diabetes, mastitis during lactation period. Further exclusion criteria were use of proton pump inhibitors, H2 receptor antagonists, antidepressants, narcotics, anticholinergic medications, laxatives, regular consumption of commercially available prebiotic- or probiotic-supplemented products, anti-diarrhea and antibiotics medications within 30 days prior to collection of the milk samples.

The project was approved by the Research Ethics Committee of the School of Pharmaceutical Sciences, University of São Paulo, São Paulo, Brazil on 27th May 2014 and by the Research Ethics Committee of the University Hospital, University of São Paulo, São Paulo, Brazil on 7th July 2014 -CAAE: 27247614.6.0000.0067. The selected women were invited to participate in the study and enrolled after signing the Written Informed Consent Form.

After the screening, two meetings were scheduled at day 7 (±3) and day 30 (±4) after delivery (Figure S1). During the first meeting, a structured questionnaire was conducted to collect data on age, family income, number of children, use of supplements/medicines during pregnancy/lactation, bowel frequency, alcohol consumption, smoking, pre-gestational and perinatal anthropometric data (weight and height) and information about the newborn. In addition, the first 24 h food recall (24 h-day 7) for the maternal diet record was performed.

On day 30 (±4) after delivery, a second meeting was held including collection of human milk at the University Hospital. In the first meeting the volunteers had a medical appointment in addition to the research meeting. Due to the longer available time of the volunteers, we chose to collect milk on day 30. To reduce the intra-personal variability in food consumption assessment, an additional 24 HR (24 h-day 30) was performed. Additionally, a quantitative food frequency questionnaire (QFFQ) relative to the pregnancy period was performed.

### 2.2. Maternal Diet Records

The maternal diet records during pregnancy were estimated by QFFQ, developed and validated for pregnant women in Brazil to encompass the entire period of pregnancy [23]. The QFFQ includes 85 items of usual foods and recipes for this population, the consumption frequency (daily, weekly, monthly or during the gestational period), the number of times the participant consumes a particular food item, the median portion (in household measures and in g/mL), and the size of the portion of each participant. The 24 h comprised information about the food intake during the last 24 h prior to the interview, data on foods and beverages currently consumed, including preparation, and information on weight and portion size in grams, milliliters or home measures. The 24 h were performed through the Multiple-Pass Method, in which the respondent is guided through five steps (quick list, forgotten foods list, time and occasion, detail and review, final probe) in a standardized process, which helps to maintain the individual interested and engaged in the interview, and helps them to remember all the items consumed [24], and when necessary, changes were made to adequate the Brazilian food reality. The food surveys were conducted by trained nutritionists.

It is noteworthy that differences in the methods used in the present study to investigate the usual intake during pregnancy (QFFQ) and during lactation (24 h) was due to the study design, which was planned to start after delivery. This was due to the inclusion criteria, which included only women who had vaginal delivery in order to avoid adding more variables in the study. Therefore, since we could not predict the mode of delivery of the volunteer, it was unfeasible to collect 24 h from the period of pregnancy.

The Nutrition Data System for Research (NDSR, version 2014, Center for Nutritional Coordination, University of Minnesota, Minneapolis, MN, USA) was used to convert the information from the food intake report from the QFFQ and the two 24 h to nutrients, for analysis of nutrients consumed during the pregnancy and lactation periods studied. Since this software uses the American food composition database developed by the United States Department of Agriculture (USDA), the adequacy of nutritional values was checked using the Brazilian Table of Food Composition [25]. In addition, folate and iron values were adjusted considering the mandatory fortification of prevailing wheat and corn flours in Brazil since 2004. In case of nutritional supplement used by the volunteers, these were added to the consumption data.

The nutrient data from 24 h were processed in the Multiple Source Method program, in order to estimate the usual dietary intake of the volunteer during the lactation period [26]. Afterwards, the nutrients intake from QFFQ and 24 h data was normalized using the residual method to standardize for energy intake [27].

We reinforce that, as a cross-sectional study, no food or supplement were supplied by the researchers to the volunteers during the study and that their diets were consumed *ad libitum*.

### 2.3. Human Milk Samples Collection

Human milk samples were taken from volunteers at 30 (±4) days after delivery, at the University Hospital of the University of São Paulo, according to Martín et al. [7] and Jost et al. [28]. The women were informed to clean their nipple and surrounding area with chlorhexidine 1% to reduce the presence of skin bacteria. Next, the first drops (approximately 200 µL) were discarded and the milk samples were collected in a sterile tube by manual pressure using sterile gloves. All the samples were kept on ice for up to 4 h; the samples were then aliquoted and stored at −80 °C for later DNA extraction.

### 2.4. DNA Extraction

Total DNA was extracted with the QIAamp DNA Mini Kit (QIAGEN, Hilden, Germany), according to the manufacturer’s protocol for Gram-positive bacteria, with slight adaptations. Briefly, 1.5 mL of human milk sample was centrifuged at 15,700× *g* for 15 min to pellet prokaryotic cells. The supernatant was discarded, and the pellet washed in 1000 µL of Tris EDTA (10 mM Tris-HCl [pH 7.5], 1 mM EDTA [pH 7.6]) buffer. The suspension was centrifuged at 15,700× *g* for 15 min. The samples were lysed in 200 µL of TELS (20 mg/mL lysozyme:1 M Tris-HCl [pH 7.5], 0.5 M EDTA [pH 8.0], 20% sucrose) buffer. Next, it was incubated for 60 min at 37 °C. The next steps followed the manufacturer’s instructions. DNA quality and concentrations were determined using a Nanodrop ND-1000 (Thermo Fisher Scientific, Waltham, MA, USA).

Additionally, to control for bacterial DNA contamination that may derive from reagents in the DNA extraction kit, we extracted DNA from a tube only containing the reagents from the DNA extraction kit (“Blank”), and moreover made a non-template PCR control (NTC) alongside the milk samples.

### 2.5. Amplicon Sequencing

The PCR-based library formation was performed by a nested PCR. The primers are listed in the Table 1.

The PCR reactions were carried out in a 25 μL mixture (final volume), containing 500 nM (for the first round) or 200 nM (for the second round) of each primer pair, 0.2 mM dNTPs (Thermo Fisher Scientific, Waltham, MA, USA), 0.5 units Phusion high fidelity DNA polymerase (Thermo Fisher Scientific, Waltham, MA, USA), 1 × Phusion Green HF buffer (Thermo Fisher Scientific), and 10 µL of sample DNA (for the first round) or 2 µL of PCR product from the first amplification (for the second round). Cycling conditions were: 98 °C for 30 s, followed by 40 cycles for the first or 15 cycles for second round of amplification, consisting of 98 °C for 5 s, 56 °C for 20 s, and 72 °C for 20 s. Negative controls with buffer from the DNA extraction kit controls were included in the PCR runs.

The PCR products were purified using Agencourt AMPure XP beads (Beckman Coulter, Danvers, MA, USA). The amplicon concentration was quantified using the Quant-iT PicoGreen dsDNA assay kit (Thermo Fisher Scientific, Waltham, MA, USA). Subsequently, samples were pooled using 3.6 ng of each. The pooled samples were loaded onto the Illumina MiSeq clamshell style cartridge kit v2 at 500 cycles, for paired-end 250 sequencing at the final concentration of 4 pM on an Illumina MiSeq (Illumina^®^, San Diego, CA, USA).

### 2.6. 16SrRNA Gene Sequence Processing

For 16S rRNA gene data analysis, generated sequences were analyzed using Quantitative Insights Into Microbial Ecology (QIIME) v1.9.1 with default settings. Chimera checking was performed using UCHIME69 and de novo Operational Taxonomic Units (OTU)-picking was performed using UCLUST70 with 97% sequence similarity. Representative sequences were assigned taxonomy against the Greengenes database v13_871 using the RDP-classifier [31].

Subsequent analyses were performed with the R version 3.4.3 using the metagenomeSeq [32], phyloSeq [33], vegan [34], and ggplot2 [35] packages. Data were filtered for low abundance of OTUs by removal of OTUs present in fewer than 3 of all the samples and with a relative abundance higher than 0.5% across all OTUs.

After quality filtering an average of 33,217 high-quality 16S rRNA gene sequences per sample remained. Reads were classified into 334 OTUs covering a median of 95 percent of total number of species across samples (min-max: 92–98).

The global microbiota composition in the milk samples was distinct from the analytical controls (PERMANOVA, controls vs. milk samples, *p* = 0.001) and we found no significant correlations between abundances at the Genus level in the analytical controls versus milk samples (Figure S2). Thus, we considered that any eventual contamination included during sample preparation was negligible to the remaining analysis.

### 2.7. Statistical Analysis

Statistical analysis was performed on data filtered based on effective sample sizes, where samples were not included if they had fewer than 10,000 or more than 100,000 OTUs.

All statistical analyses were performed using R (R version 3.4.3, Vienna, Austria). For differences in the human milk microbiota composition, PERMANOVA was performed using the Adonis function in vegan [34] with weighted UniFrac distance. For each variable, 999 permutations were made.

Associations between nutrient intake and microbial taxa were conducted using Spearman rank correlation tests.

For the clustering analysis, samples were clustered based on relative genus abundances, and using weighted UniFrac distance via partitioning around medoid (PAM) clustering algorithm in the R package “cluster” [36]. Optimal number of clusters was estimated using the prediction strength (PS) [37]. Alpha-diversity analyses were performed after applying rarefactions (10,000 sequences/sample) to standardize sequence counts (vegan package). After checking for normality, *t*-test or Wilcoxon signed ranks test was used for parametric or non-parametric data to compare clusters and variables.

A false discovery rate *p* value ≤ 0.01 was considered as significant for Spearman’s correlation tests. A standard *p* value < 0.05 was considered as significant for all other analyses.

### 2.8. Data Deposition and Materials Sharing

Sequence data have been deposited in the National Center for Biotechnology Information (NCBI) under the project number PRJNA479106 (https://www.ncbi.nlm.nih.gov/Traces/study/?acc=SRP151896) and are available under SRA accession SRP151896.

## 3. Results

Two hundred forty-three lactating women were assessed for eligibility for inclusion in the study. Of this group, 12 refused to participate, 10 did not meet the inclusion criteria, 119 did not attend all the meetings, and 8 volunteers were excluded during analysis as their samples did not yield sufficient sequencing depth. Thus, milk collected at 1-month post-partum from 94 volunteers was included in the analysis. The clinical and demographic characteristics of these volunteers are shown in Table 2.

### 3.1. The Milk Microbiota of Brazilian Mothers is Dominated by Streptococcus, Staphylococcus, and Corynebacterium

**Figure 1:**
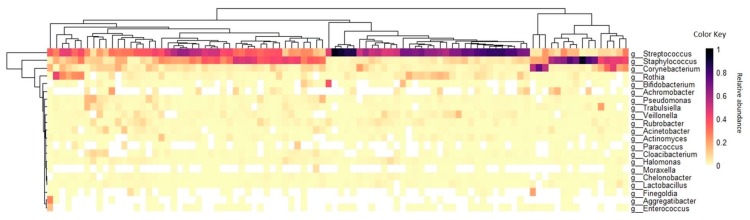
Relative abundance heatmap of the most abundant bacterial genera identified in human milk samples. Legend: The rows present the genera identified in at least 90% of the samples or with maximum relative abundance higher than 0.05. Column represents the milk samples from volunteers at 30 (±4) days after delivery.

The characterization of the microbiota in the milk of 94 volunteers showed that *Firmicutes* was the most abundant phylum (a mean average of 70%), followed by *Actinobacteria* (14.5%), *Proteobacteria* (14%), and *Bacteroidetes* (1%) (Figure S3). A total of 85 genera were identified across human milk samples. However, we found a core consisting of three genera present in all samples: *Streptococcus*, *Staphylococcus*, and *Corynebacterium* (relative mean abundances of 42%, 22%, and 7%, respectively), and ten genera present in at least 90% of the samples: *Rothia* (relative mean abundance of 0.67%), *Veillonella* (0.41%), *Rubrobacter* (0.38%), *Pseudomonas* (0.32%), *Halomonas* (0.28%), *Trabulsiella* (0.19%), *Chelonobacter* (0.14%), *Acinetobacter* (0.11%), *Actinomyces* (0.08%) and *Lactobacillus* (0.06%)*. Bifidobacterium*, an important genus reported to influence infant health, was present in 78% of the samples and in relative mean abundances of 1% (Figure 1) and family *Bifidobacteriaceae* was detected in 84% of all samples.

### 3.2. Maternal Diet during Pregnancy Modulates Milk Microbiota Composition while the Diet during Lactation Results in Minor Changes in Taxa Abundance

We evaluated the effect of the maternal diet by one QFFQ relative to the pregnancy period and two 24 h to estimate intake of nutrients during the first month of lactation. To identify associations between nutrients and human milk bacteria community, PERMANOVA tests were performed using the weighted UniFrac distance for each nutrient, with each nutrient as a dependent variable. Significant differences in microbiota composition were found for the intake levels of vitamin C during pregnancy (*p* = 0.029) (Table S1). No significant differences were found for nutrients intake from the lactation period. The bacteria composition of human milk at 1-month post-partum was found not to be significantly affected by maternal ethnicity (race), age, socioeconomic level, number of children, duration of pregnancy, antibiotic use during pregnancy/delivery, Body Mass Index (BMI) before pregnancy, weight gain during pregnancy, anesthesia at delivery, BMI at day 30 after delivery, sporadic offering of infant formula, or infant weight gain during the first month after birth (Table S1).

Spearman’s correlations identified further associations between nutrients and bacterial genera (Figures S4 and S5). Correlation between nutrients and bacterial genera differed in relation to maternal diet estimated during pregnancy and lactation period. There were 17 significant correlations detected between diet assessed during pregnancy and the milk genera detected, while diet during the lactation period showed 41 significant correlations with the milk bacterial genera detected, although most of the correlations during the lactation period were found for genera present at low relative abundance in the samples. (Figures S4 and S5). Particularly, the intake of vitamin C during pregnancy was positively correlated with the presence of *Staphylococcus* genus (rho = 0.25, *p* = 0.01). For the lactation period, positive correlations were found between *Bifidobacterium* and intake of polyunsaturated fatty acids (PUFAs) (rho/coefficient = 0.29, *p* = 0.005) and linoleic acid (rho/coefficient = 0.27, *p* = 0.007). In addition, negative correlations were found between intake of sugars and *Pseudomonas* (rho/coefficient = −0.27, *p* = 0.0085) and positive correlations were found between intake of vitamin B9 and *Pseudomonas* (rho/coefficient = 0.29, *p* = 0.0053). The intake of complex B vitamins, particularly B1 (rho/coefficient = −0.38, *p* = 0.0002), B2 (rho/coefficient = −0.27, *p* = 0.0084), and B9 (rho/coefficient = −0.43, *p* = 0.00001) were negatively correlated with *Enterococcus*.

**Figure 2:**
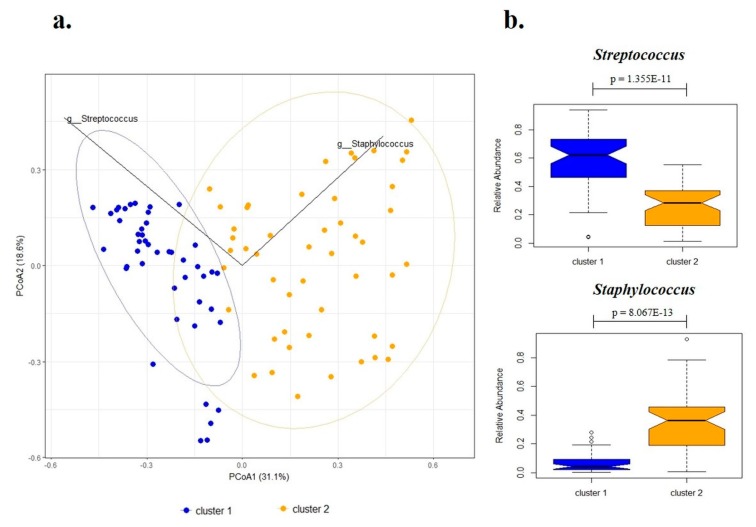
Clusters identified in human milk samples. (**a**) Principal Coordinate Analysis (PCoA) clustering of human milk samples driven by *Streptococcus* (cluster 1) and *Staphylococcus* (cluster 2). (**b**) Relative abundance of bacterial taxa characteristic in each cluster. Box-plot representing the interquartile range (IQR) and the line inside represents the median. Small circles denote outliers. Legend: Mann-Whitney test was performed to compare the relative abundance between the clusters.

Next, we grouped the samples in clusters based on microbiota profile similarities. The highest prediction strength using weighted UniFrac was obtained for two clusters (prediction strength = 0.78 for 2 clusters, Figure S6). Principal Coordinate Analysis (PCoA) indicated that these two clusters were driven by *Streptococcus* (cluster 1) and *Staphylococcus* (cluster 2) (Figure 2).

Other differences between the clusters were found for the genera *Corynebacterium* [1.8% (cluster 1) vs. 4.3% (cluster 2); *p* = 0.007], *Pseudomonas* [0.2% (cluster 1) vs. 0.7% (cluster 2); *p* = 0.003], *Rothia* [2.6% (cluster 1) vs. 0.3% (cluster 2); *p* = 0.00001] and *Trabulsiella* [0.1% (cluster 1) vs. 0.3% (cluster 2); *p* = 0.006]. *Bifidobacterium* was detected in slightly higher amounts in cluster 2, but this difference was not statistically significant.

No differences were found between the clusters in term of genus richness (Chao1 index), and alpha-diversity (Shannon index) (Figure S7). Clusters did not differ with respect to maternal ethnicity, age, socioeconomic level, number of children, duration of pregnancy, antibiotic use during pregnancy/delivery, BMI before pregnancy, weight gain over pregnancy, anesthesia at delivery, BMI at day 30 after delivery, sporadic offering of infant formula or infant weight gain over 30 days after birth (Table S2).

**Figure 3:**
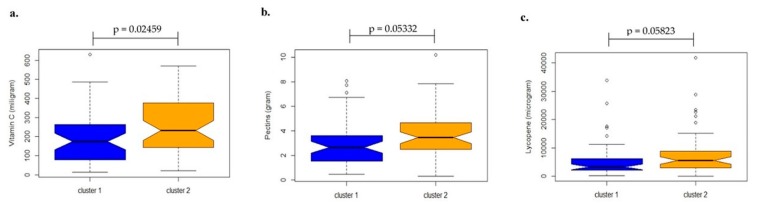
Distribution of nutrients intake from pregnancy, by clusters identified in human milk samples. Vitamin C (**a**), pectins (**b**), and lycopene (**c**) intake estimated by a quantitative food frequency questionnaire (QFFQ) for pregnancy, in each cluster. Legend: The box-plot is representing the interquartile range (IQR) and the line inside represents the median. Small circles denote outliers. Mann-Whitney test was performed to compare the values between the clusters.

We found differences in vitamin C intake by the mothers during pregnancy based on the milk microbiome clusters (median in cluster 1 = 175 mg/d, cluster 2 = 232 mg/d, *p* = 0.0249; Figure 3a). The vitamin C intake was higher in cluster 2, driven by the *Staphylococcus* genus. We also found trends towards higher levels of intake of pectin (Figure 3b, *p* = 0.053) and lycopene (Figure 3c, *p* = 0.058) during pregnancy in cluster 2, even though this trend was not statistically significant. No statistical differences were found for nutrient intake during the first month of lactation based on milk microbiome clusters (Table S2).

## 4. Discussion

The importance of human milk on the infant’s health is well documented. Over the last years, studies have focused on the potential role of human milk in the development of the immune system during infancy and childhood [38], as well as on modulating the infant gut microbiota [39].

Our results revealed that 4 phyla-*Firmicutes*, *Actinobacteria*, *Proteobacteria*, and *Bacteroidetes* are dominating in the human milk microbiota on day 30 after delivery. In line with previous studies *Firmicutes* and *Proteobacteria* were the most abundant phyla [4,40]. At the genus level, *Streptococcus* (*Firmicutes*) and *Staphylococcus* (*Firmicutes*) were identified as the most abundant genera, followed by *Corynebacterium* (*Actinobacteria*). We found more than 80 genera showing that the human milk microbiota hosts a diverse set of bacteria.

Several studies have identified *Staphylococcus* and *Streptococcus* as the most abundant genera in human milk [9,41]. Since these genera are typical inhabitants of the skin and oral cavity, our findings are consistent with the hypothesis of a retrograde flux of bacteria present on the mother’s skin/infant’s oral cavity to the human milk, enabling these bacteria to contribute to the composition of the human milk microbiota [42]. Previous studies have reported a human milk core microbiome consisting of several genera [4,9]. However, we have detected five genera (*Staphylococcus*, *Streptococcus, Corynebacterium*, *Pseudomonas* and *Lactobacillus*) previously reported and additionally seven genera present in at least 90% of the studied individuals.

Since *Bifidobacterium* and *Lactobacillus* are highly present in the gut microbiota of breast-fed infants, we expected to find these genera in relative high abundance also in the milk. Surprisingly, the average relative abundances ranged from just 0.1 to 1% for *Bifidobacterium* and from 0.1 to 0.3% for *Lactobacillus*. This is less than reported in previous studies by Urbaniak et al. [40] who reported around 3% of *Lactobacillus*, and Murphy et al. [4] who reported around 2% of *Bifidobacterium* and *Lactobacillus* in the first weeks after delivery. This differences could indicate a broader human milk core microbiota, of point at inherent differences in the human milk microbiota from Brazilian women, as well as differences in methodological factors, including DNA extraction procedures, PCR bias, use of different primer sets assessing different *16S rRNA* gene regions, as well as depth of sequencing of the study [28].

The microbiota from all samples was classified into two clusters driven by *Streptococcus* and *Staphylococcus*. This is in line with previous studies of human milk samples, although the two genera were reported with higher relative abundance [9,11,40]. Indeed, a recently published systematic review of the human milk microbiota identified the predominance of *Streptococcus* and *Staphylococcus* in 12 analyzed studies, suggesting that these genera may be universally predominant in human milk, regardless of differences in geographic location or analytic methods [8].

Interestingly, Li et al. [43] reported 3 clusters based on family profiles of human milk microbiota in samples collected from Taiwan and six regions of mainland China. The clusters were driven by the *Streptococcaceae*, *Staphylococcaceae* or *Pseudomonadaceae* families showing that taxonomic differences in clusters do occur.

The impact of *Staphylococcus* and *Streptococcus* on the infant’s health is not completely clear, but it is known that these genera are present in the infant’s gut, especially during the first weeks [1,44,45]. Studies suggest that *Staphylococcus* and *Streptococcus*, as well as other facultative aerobic species are the initial gut colonizers. These species contribute to the consumption of oxygen from the infant’s gut, providing a suitable environment for the colonization of strict anaerobic species, such as *Bifidobacterium*, *Bacteroides*, and *Clostridium* [46].

In our study, we did not find any effect on the two bacterial clusters dominated by *Streptococcus* and *Staphylococcus* by maternal socioeconomic/clinical information or nutrient intake during the lactation period. However, weak positive correlations were found between the intake of polyunsaturated/linoleic fatty acids during the lactation period and *Bifidobacterium* in the milk, genus considered of importance for the infant’s health [47,48]. In fact, previous studies have reported a relation between the PUFA intake and milk composition [15,49]. Recently, the new consensus statement on the definition and scope of prebiotics includes PUFA as a prebiotics [50]. According to Gibson et al. [50] the mechanisms involved in the prebiotic property of PUFA are related to the conversion into the conjugated linoleic acid (CLA) and conjugated linolenic acid (CLnA). In line with the positive correlations between PUFA/linoleic acid and *Bifidobacterium* found in this study, the conversion of PUFA into CLAs is reported to be followed by growth of *Bifidobacterium* [51].

During the lactation period, we also found negative correlations between intake of thiamin, riboflavin, and folate and the *Enterococcus* genus. The *Enterococcus* genus has been found in human milk samples in previous studies [4,11], as well as in the infant gut [1]. We predicted positive correlation between these vitamins and *Enterococcus*, since most *Enterococci* require complex B vitamins in their metabolism for maximal growth [52]. However, this was not the case. The fact that vitamins usually are bound to other compounds, predominantly proteins, might suggest that maternal factors regulating protein secretion are more likely to affect milk levels of these vitamins than the fluctuating maternal intake [52].

Although we did not find any statistically significant differences among maternal diet in the lactation period and the clusters, we found significant differences between the level of vitamin C intake during pregnancy and the clusters, with higher levels of vitamin C intake in cluster 2, driven by the *Staphylococcus* genus. This finding is in accordance with the results obtained by the Spearman’s correlation test, indicating a positive correlation between vitamin C intake during pregnancy and the *Staphylococcus* genus.

In contrast to our findings, vitamin C has been reported to inhibit the growth of *Staphylococcus* [53]. However, most of the studies investigated the impact of high concentrations of this vitamin (more than 1 g/L), which is above the physiologic concentrations found in human milk samples (ca. 0.04 g/L) [53,54]. On the other hand, other studies performed with citrus fruit juice, which are rich in vitamin C, showed no negative effect on abundance of *Staphylococcus* [55,56].

Although the role of vitamin C in *Staphylococcus* metabolism remains unclear, both have been linked to the development of the immune system. According to Hoppu et al. [57] higher vitamin C levels in breast milk was associated with reduced risk of atopy in high-risk infants. At the same time, non-allergic children were shown to have higher counts of *Staphylococcus* in feces, when compared to allergic counterparts [58,59]. In this context, Salminen et al. [60] suggested that *Staphylococcus aureus*, traditionally considered as harmful, but frequently isolated from infant feces, may aid in educating the coevolving immune system. Thus, the link between vitamin C and *Staphylococcus* abundance warrant further investigation.

We also observed a tendency towards higher maternal intakes of pectin and lycopene during pregnancy in cluster 2. It is noteworthy to mention that vitamin C, pectin, and lycopene are compounds typically found together in citrus fruits [61]. In this context, higher maternal consumption of citrus fruit, as well as vegetables and β-carotene during pregnancy were protective against the development of eczema in the offspring, in line with the positive effects of vitamin C and *Staphylococcus* on the immune system [62].

Curiously, fruits and fruit juices have been reported as craved foods in pregnancy by several studies [63]. The fluctuations of hormones and the instinct to protect the embryo from toxic substances and pathogens have been hypothesized to be the reason for food cravings, and/or aversions [63,64]. We speculate that this behavior commonly found in pregnant women could be, not only related to the physiology of pregnancy, but also a contributor for the bacterial community structure in the human milk.

In a previous study, Wu et al. [18] demonstrated the association between the long-term dietary patterns and gut enterotypes; particularly protein and animal fat were associated with *Bacteroides* and carbohydrates with the *Prevotella* enterotype. Our results again highlight the effects of long-term over short-term dietary habits, herein represented by the maternal intake during pregnancy.

## 5. Study Limitation and Future Directions

It is a limitation of this study that the QFFQ for pregnancy was collected once, at day 30 post-partum. Follow-up studies considering several points of food records collection, as well as daily longitudinal sampling or more points of sampling over the lactation period would allow us to test directly for associations between dietary components and microbiome composition within each participant and the identification of microbiota patterns over time.

In addition, the correlations between maternal nutrients intake and milk microbiota were done by tests with unadjusted *p*-values, and as such this should be taken as an observational study. Interventional trials would be interesting to test the impact of certain foods or nutrients, (e.g., vitamin C, polyunsaturated fatty acids) on the milk microbiota composition, to validate our findings.

The lack of reports on the role of nutrients in the metabolism of different bacteria in human milk, and the limited knowledge regarding the availability of macro and micronutrients in the human milk for the bacterial use complicate data interpretation. Thus, in vitro studies with bacteria isolated from human milk inoculated in human milk/culture media containing different concentrations of nutrients are warranted.

## 6. Conclusions

In conclusion, our results demonstrate that the maternal diet influences the human milk microbiota, and that the effects of the maternal diet seem to be different for diet during pregnancy and during the lactation period. The diet during pregnancy has a stronger impact on the bacterial community structure compared to the diet during the first month of lactation, where specific nutrients in contrast showed to differentially affect abundances of specific bacterial genera. For dietary patterns during pregnancy, intake of vitamin C was clearly associated with the composition of the human milk microbiota. Minor changes in genus abundances were found to correlate with the maternal short-term food intake, represented by the lactation period. Specifically, PUFA/linoleic fatty acid correlated positively with the relative abundance of the genera *Bifidobacterium* in human milk, which may play a role in the composition of the developing infant gut microbiota.

## Figures and Tables

**Table 1:** Primers used for the PCR-based library formation.

	Primer	Sequence	Reference
PCR 1st round	341F	CCTAYGGGRBGCASCAG	Yu et al. [29]
	806R	GGACTACHVGGGTWTCTAAT	
PCR 2nd round	515F	AATGATACGGCGACCACCGAGATCTACAC-NNNNNNNN-GTGTGCCAGCMGCCGCGGTAA*	Caporaso et al. [30]
	806R	CAAGCAGAAGACGGCATACGAGAT-NNNNNNNNNNNNAGTCAGTCAGCCGGACTACHVGGGTWTCTAAT*	

* These primers are targeting the 16S rRNA gene’s variable region (V4), where “N” denotes index sequence for multiplexing.

**Table 2:** Clinical and demographic characteristics of the volunteers included in the analysis (*n* = 94).

Variables	Values
**Maternal age (years)**	27 (22.3–29)
**Race**	
Black/Brown	41 (44)
White	53 (56)
Family income estimated (USD/month) *	462.5 (462.5–770.39)
**Number of children**	
1	32 (34)
2 or more	62 (66)
**Duration of pregnancy (weeks)**	39 (38–40)
**Maternal antibiotic treatment**	
Number with treatment during pregnancy	26 (28)
Number with treatment during delivery	40 (42)
**Alcohol drinking during pregnancy**	5 (5.3)
**Smoking during pregnancy**	9 (9.5)
**BMI before pregnancy (kg/m^2^)**	23.0 (21.1–24.6)
**Maternal weight gain over pregnancy (kg)**	11.4 (8.7–14.0)
**Anesthesia**	
No anesthesia	63 (67.1)
Pudendal block	3 (3.1)
Epidural	5 (5.3)
Spinal	23 (24.5)
**BMI at day 30 after delivery (kg/m^2^)**	23.8 (22.1–26.7)
**Infant diet at day 30 after delivery**	
Exclusively breast milk	79 (83)
Breast milk + formula **	16 (17)
**Infant weight gain over 30 days after birth (g)**	1160 (890–1470)

Data presented as median (interquartile range) or *n* (%). BMI: body mass index (kg/m^2^). * Family income estimated by Brazilian Economic Classification Criteria (Brazilian Criteria) 2016. ** Frequency of offering formula ≤ 3 times/day.

## Supplementary Materials

The following are available online at https://www.mdpi.com/2076-2607/7/11/502/s1, Table S1. *p* values obtained by using PERMANOVA tests for weighted UniFrac distances for each variable. Table S2. *p* values obtained by using t tests (parametric) or Mann-Whitney test (non-parametric) for comparisons between clusters and continuous data or Chi-square test for categorical data. Figure S1. Study design for data and milk samples collection. Legend: 24 h: 24 h food recall; HM: Human milk; QFFQ: Quantitative food frequency questionnaire. Figure S2. Comparison of bacterial profile between human milk samples and controls. Legend: To verify the microbiota from reagents in either the DNA extraction kit or from PCR, we extracted the DNA of an empty tube containing the reagents from the DNA extraction kit (“Blank”) and a no template control (“NTC”) for PCR alongside the milk samples. Data presented as genera relative abundance normalized by using z-score in columns. The bacterial profile from milk samples differs from those of controls (*p* = 0.001 by PERMANOVA test comparing controls vs. milk samples). Figure S3. Relative abundance of the bacterial phyla identified in human milk samples. Box-plot representing the interquartile range (IQR) and the line inside represents the median. Small circles denote outliers. Figure S4. Correlation between maternal diet during pregnancy and human milk bacterial genera. Legend: Columns correspond to estimated nutrient intake measured by a quantitative food frequency questionnaire; rows correspond to bacterial genus. Red and blue denote positive and negative association, respectively. The intensity of the colors represents the strength of the association between the genus abundance and nutrients as measured by Spearman’s correlations. Asterisks indicate the associations that are significant after adjusting for multiple comparisons (*p* ≤ 0.01). Rows are clustered by Euclidean distance. Figure S5. Correlation of maternal diet during lactation and human milk bacterial genera. Legend: Columns correspond to estimated nutrient intake measured by two 24 h food recalls; rows correspond to bacterial genus. Red and blue denote positive and negative association, respectively. The intensity of the colors represents the degree of association between the genus abundance and nutrients as measured by Spearman’s correlations. Asterisks indicate the associations that are significant for multiple comparisons (*p* ≤ 0.01). Rows are clustered by Euclidean distance. Figure S6. Optimal number of clusters displayed using the prediction strength measure and the weighted UniFrac distance. Legend: The panel shows that the data best separated into two clusters by the partitioning around medoid (PAM) method. Figure S7. Richness and alpha diversity values of milk samples, by clusters. The richness is measured for Chao1 (a), and alpha diversity for Shannon (b). Legend: Operational Taxonomic Unit table for each measure was rarefied to 10,000 reads. The box-plot is representing the interquartile range (IQR) and the line inside represents the median. Black-filled small circles denote the distribution of measures for each sample; white-filled small circles denote outliers. Mann-Whitney test was performed to compare the values between the clusters.
