# Supplementary material for: The Human Milk Microbiota is Modulated by Maternal Diet
Source: Microorganisms. 2019 Oct 29;7(11):502. doi: 10.3390/microorganisms7110502 (PMC6920866; doi:10.3390/microorganisms7110502)
Supplement: Supplementary file 1 [file microorganisms-07-00502-s001.zip › Table_S1.docx]

| **Variable** | **p values (weighted UniFrac)** |
| --- | --- |
| **Clinical and demographic characteristics** | |
| Maternal age | 0.312 |
| Race | 0.490 |
| Socioeconomic level | 0.231 |
| Number of children | 0.389 |
| Duration of pregnancy | 0.364 |
| Maternal antibiotic treatment (pregnancy) | 0.115 |
| Maternal antibiotic treatment (delivery) | 0.666 |
| Alcohol drinking during pregnancy | 0.278 |
| Smoking during pregnancy | 0.872 |
| BMI before pregnancy | 0.214 |
| Maternal weight gain over pregnancy | 0.367 |
| Anesthesia | 0.318 |
| BMI at day 30 after delivery | 0.563 |
| Infant diet at day 30 after delivery | 0.474 |
| Infant weight gain over 30 days after birth | 0.555 |
| **Nutrients intake, during pregnancy** | |
| Energy | 0.945 |
| Total carbohydrates | 0.962 |
| Added sugars | 0.523 |
| Total proteins | 0.777 |
| Total fat | 0.623 |
| Total saturated fatty acid | 0.369 |
| Total monounsaturated fatty acid | 0.698 |
| *continuation* |  |
| Total polyunsaturated fatty acid | 0.727 |
| Linoleic fatty acid (18:2 n-6) | 0.770 |
| Linolenic fatty acid (18:3 n-3) | 0.640 |
| Total trans fatty acid | 0.623 |
| Animal protein | 0.404 |
| Vegetable protein | 0.723 |
| Cholesterol | 0.777 |
| Total dietary fiber | 0.449 |
| Soluble fiber | 0.473 |
| Insoluble fiber | 0.698 |
| Pectin | 0.295 |
| Vitamin A | 0.782 |
| Vitamin D | 0.464 |
| Vitamin E | 0.792 |
| Vitamin K | 0.716 |
| Vitamin C | **0.029** |
| Vitamin B1 (thiamin) | 0.761 |
| Vitamin B2 (riboflavin) | 0.432 |
| Vitamin B3 (niacin) | 0.807 |
| Vitamin B5 (pantothenic acid) | 0.592 |
| Vitamin B6 (pyridoxin) | 0.403 |
| Vitamin B9 (folate) | 0.821 |
| Vitamin B12 (cyanocobalamin) | 0.201 |
| Calcium | 0.369 |
| Iron | 0.802 |
|  |  |
| *continuation* |  |
| Phosphorus | 0.611 |
| Magnesium | 0.624 |
| Manganese | 0.812 |
| Copper | 0.639 |
| Selenium | 0.844 |
| Sodium | 0.877 |
| Potassium | 0.187 |
| Zinc | 0.787 |
| Lutein – Zeaxanthin | 0.688 |
| Lycopene | 0.654 |
| **Nutrients intake, during lactation** | |
| Energy | 0.229 |
| Total carbohydrates | 0.794 |
| Added sugars | 0.659 |
| Total proteins | 0.842 |
| Total fat | 0.320 |
| Total saturated fatty acid | 0.618 |
| Total monounsaturated fatty acid | 0.310 |
| Total polyunsaturated fatty acid | 0.121 |
| Linoleic fatty acid (18:2 n-6) | 0.104 |
| Linolenic fatty acid (18:3 n-3) | 0.287 |
| Total trans fatty acid | 0.283 |
| Animal protein | 0.757 |
| Vegetable protein | 0.206 |
| Cholesterol | 0.508 |
|  |  |
| *continuation* |  |
| Total dietary fiber | 0.257 |
| Soluble fiber | 0.419 |
| Insoluble fiber | 0.279 |
| Pectin | 0.305 |
| Vitamin A | 0.920 |
| Vitamin D | 0.689 |
| Vitamin E | 0.624 |
| Vitamin K | 0.860 |
| Vitamin C | 0.837 |
| Vitamin B1 (thiamin) | 0.618 |
| Vitamin B2 (riboflavin) | 0.742 |
| Vitamin B3 (niacin) | 0.375 |
| Vitamin B5 (pantothenic acid) | 0.671 |
| Vitamin B6 (pyridoxin) | 0.572 |
| Vitamin B9 (folate) | 0.737 |
| Vitamin B12 (cyanocobalamin) | 0.677 |
| Calcium | 0.759 |
| Iron | 0.545 |
| Phosphorus | 0.214 |
| Magnesium | 0.360 |
| Manganese | 0.963 |
| Copper | 0.976 |
| Selenium | 0.677 |
| Sodium | 0.710 |
| Potassium | 0.126 |
|  |  |
| *continuation* |  |
| Zinc | 0.389 |
| Lutein – Zeaxanthin | 0.282 |
| Lycopene | 0.400 |
