# Supplementary material for: The Human Milk Microbiota is Modulated by Maternal Diet
Source: Microorganisms. 2019 Oct 29;7(11):502. doi: 10.3390/microorganisms7110502 (PMC6920866; doi:10.3390/microorganisms7110502)
Supplement: Supplementary file 1 [file microorganisms-07-00502-s001.zip › Table_S2.docx]

| **Variable** | **p values** | |
| --- | --- | --- |
| **Clinical and demographic characteristics** | | |
| Maternal age |  | 0.385 |
| Race |  | 0.697 |
| Socioeconomic level |  | 0.346 |
| Number of children |  | 0.159 |
| Duration of pregnancy |  | 0.090 |
| Maternal antibiotic treatment (pregnancy) |  | 0.061 |
| Maternal antibiotic treatment (delivery) |  | 1.000 |
| Alcohol drinking during pregnancy |  | 0.411 |
| Smoking during pregnancy |  | 0.893 |
| BMI before pregnancy |  | 0.764 |
| Maternal weight gain over pregnancy |  | 0.946 |
| Anesthesia |  | 0.881 |
| BMI at day 30 after delivery |  | 0.324 |
| Infant diet at day 30 after delivery |  | 0.475 |
| Infant weight gain over 30 days after birth |  | 0.141 |
| **Nutrients intake, during pregnancy** | | |
| Energy |  | 0.792 |
| Total carbohydrates |  | 0.874 |
| Added sugars |  | 0.640 |
| Total proteins |  | 0.547 |
| Total fat |  | 0.976 |
| Total saturated fatty acid |  | 0.757 |
| Total monounsaturated fatty acid |  | 0.940 |
| Total polyunsaturated fatty acid |  | 0.587 |
| *continuation* |  |  |
| Linoleic fatty acid (18:2 n-6) |  | 0.424 |
| Linolenic fatty acid (18:3 n-3) |  | 0.507 |
| Total trans fatty acid |  | 0.874 |
| Animal protein |  | 0.460 |
| Vegetable protein |  | 0.958 |
| Cholesterol |  | 0.757 |
| Total dietary fiber |  | 0.587 |
| Soluble fiber |  | 0.116 |
| Insoluble fiber |  | 0.512 |
| Pectin |  | **0.053** |
| Vitamin A |  | 0.210 |
| Vitamin D |  | 0.845 |
| Vitamin E |  | 0.908 |
| Vitamin K |  | 0.657 |
| Vitamin C |  | **0.025** |
| Vitamin B1 (thiamin) |  | 0.821 |
| Vitamin B2 (riboflavin) |  | 0.833 |
| Vitamin B3 (niacin) |  | 0.668 |
| Vitamin B5 (pantothenic acid) |  | 0.552 |
| Vitamin B6 (pyridoxin) |  | 0.507 |
| Vitamin B9 (folate) |  | 0.465 |
| Vitamin B12 (cyanocobalamin) |  | 0.845 |
| Calcium |  | 0.746 |
| Iron |  | 1.000 |
| Phosphorus |  | 0.803 |
| Magnesium |  | 0.469 |
| *continuation* |  |  |
| Manganese |  | 0.483 |
| Copper |  | 0.934 |
| Selenium |  | 0.833 |
| Sodium |  | 0.982 |
| Potassium |  | 0.145 |
| Zinc |  | 0.951 |
| Lutein – Zeaxanthin |  | 0.239 |
| Lycopene |  | **0.058** |
| **Nutrients intake, during lactation** |  |  |
| Energy |  | 0.260 |
| Total carbohydrates |  | 0.519 |
| Added sugars |  | 0.620 |
| Total proteins |  | 0.530 |
| Total fat |  | 0.953 |
| Total saturated fatty acid |  | 0.931 |
| Total monounsaturated fatty acid |  | 0.707 |
| Total polyunsaturated fatty acid |  | 0.760 |
| Linoleic fatty acid (18:2 n-6) |  | 0.714 |
| Linolenic fatty acid (18:3 n-3) |  | 0.820 |
| Total trans fatty acid |  | 0.406 |
| Animal protein |  | 0.496 |
| Vegetable protein |  | 0.513 |
| Cholesterol |  | 0.864 |
| Total dietary fiber |  | 0.066 |
| Soluble fiber |  | 0.092 |
| Insoluble fiber |  | 0.132 |
| *continuation* |  |  |
| Pectin |  | 0.231 |
| Vitamin A |  | 0.323 |
| Vitamin D |  | 0.388 |
| Vitamin E |  | 0.158 |
| Vitamin K |  | 0.688 |
| Vitamin C |  | 0.173 |
| Vitamin B1 (thiamin) |  | 0.396 |
| Vitamin B2 (riboflavin) |  | 0.702 |
| Vitamin B3 (niacin) |  | 0.161 |
| Vitamin B5 (pantothenic acid) |  | 0.520 |
| Vitamin B6 (pyridoxin) |  | 0.261 |
| Vitamin B9 (folate) |  | 0.639 |
| Vitamin B12 (cyanocobalamin) |  | 0.285 |
| Calcium |  | 0.781 |
| Iron |  | 0.217 |
| Phosphorus |  | 0.890 |
| Magnesium |  | 0.752 |
| Manganese |  | 0.452 |
| Copper |  | 0.692 |
| Selenium |  | 0.088 |
| Sodium |  | 0.425 |
| Potassium |  | 0.078 |
| Zinc |  | 0.153 |
| Lutein – Zeaxanthin |  | 0.638 |
| Lycopene |  | 0.618 |
