# Supplementary figures and images for: The Human Milk Microbiota is Modulated by Maternal Diet
Source: Microorganisms. 2019 Oct 29;7(11):502. doi: 10.3390/microorganisms7110502 (PMC6920866; doi:10.3390/microorganisms7110502)

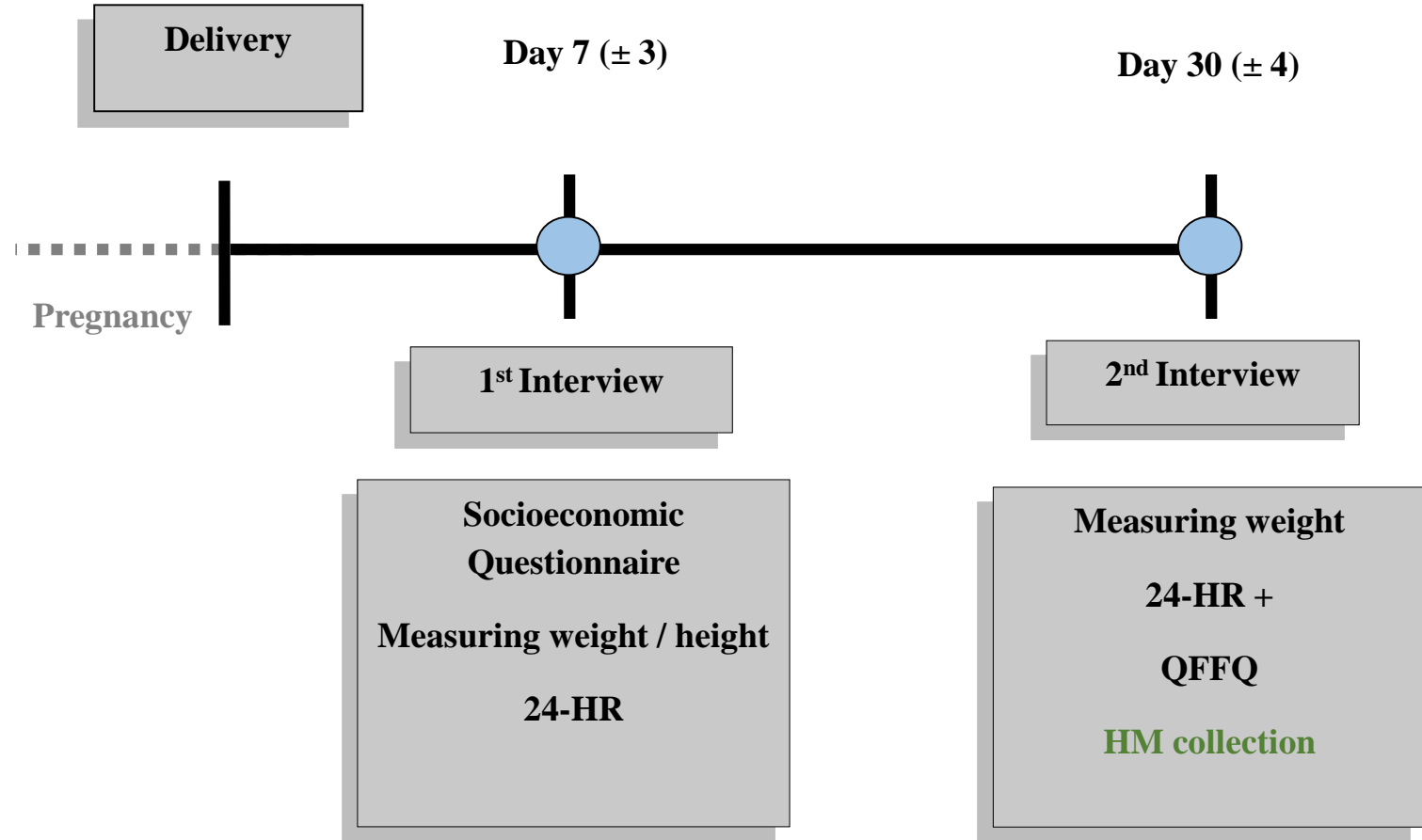

Supplement: Supplementary file 1 [file microorganisms-07-00502-s001.zip › Figure_S1.pdf]

Color Key

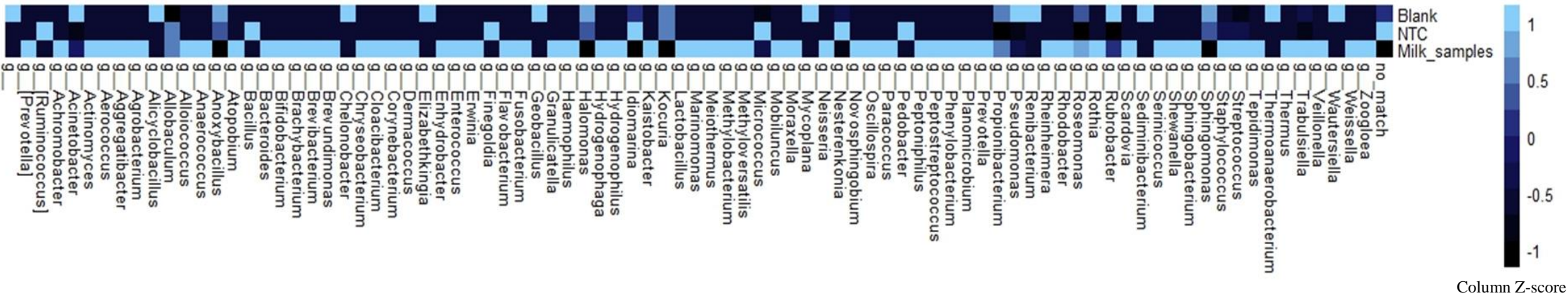

Supplement: Supplementary file 1 [file microorganisms-07-00502-s001.zip › Figure_S2.pdf]

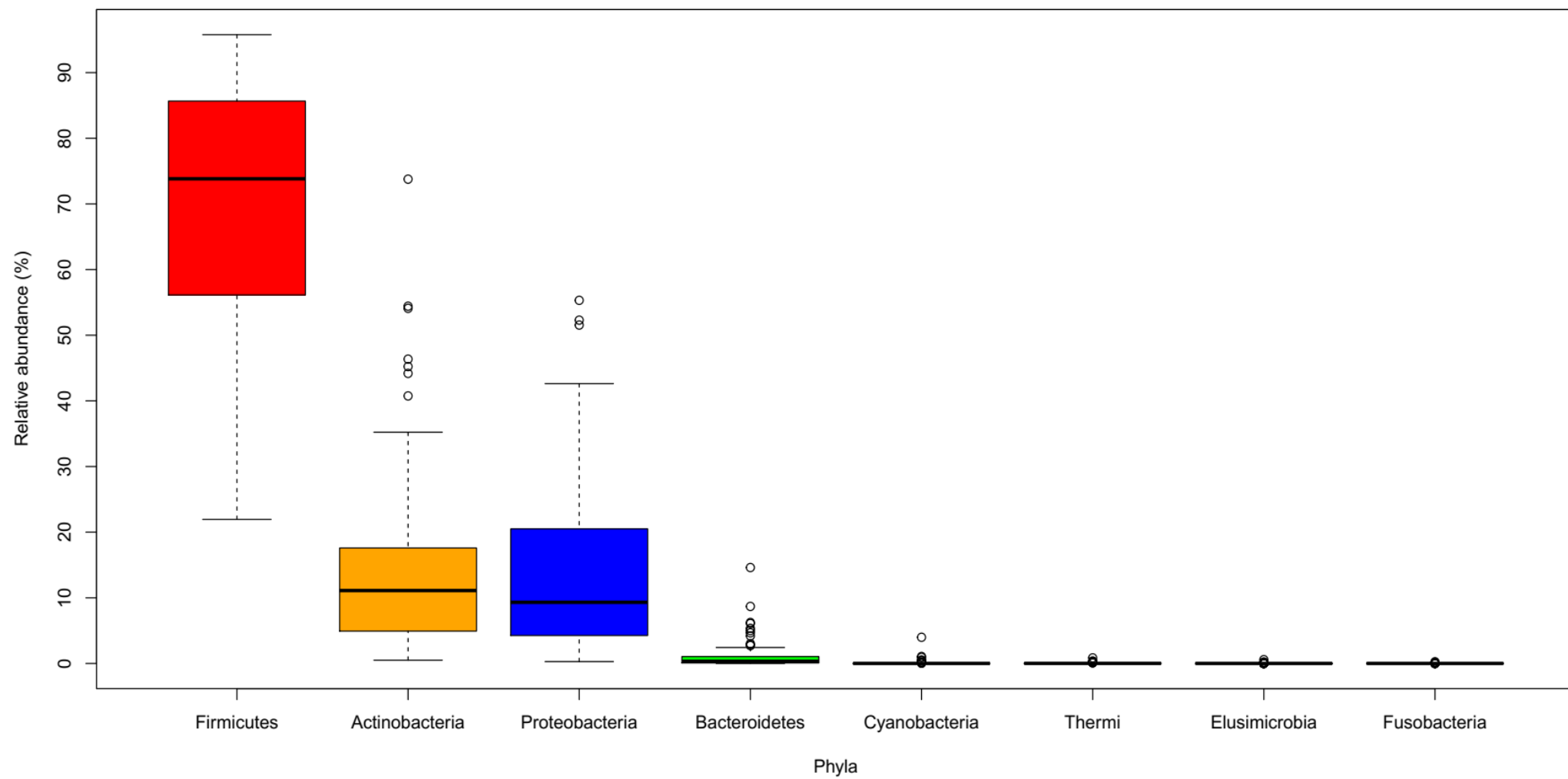

Supplement: Supplementary file 1 [file microorganisms-07-00502-s001.zip › Figure_S3.pdf]

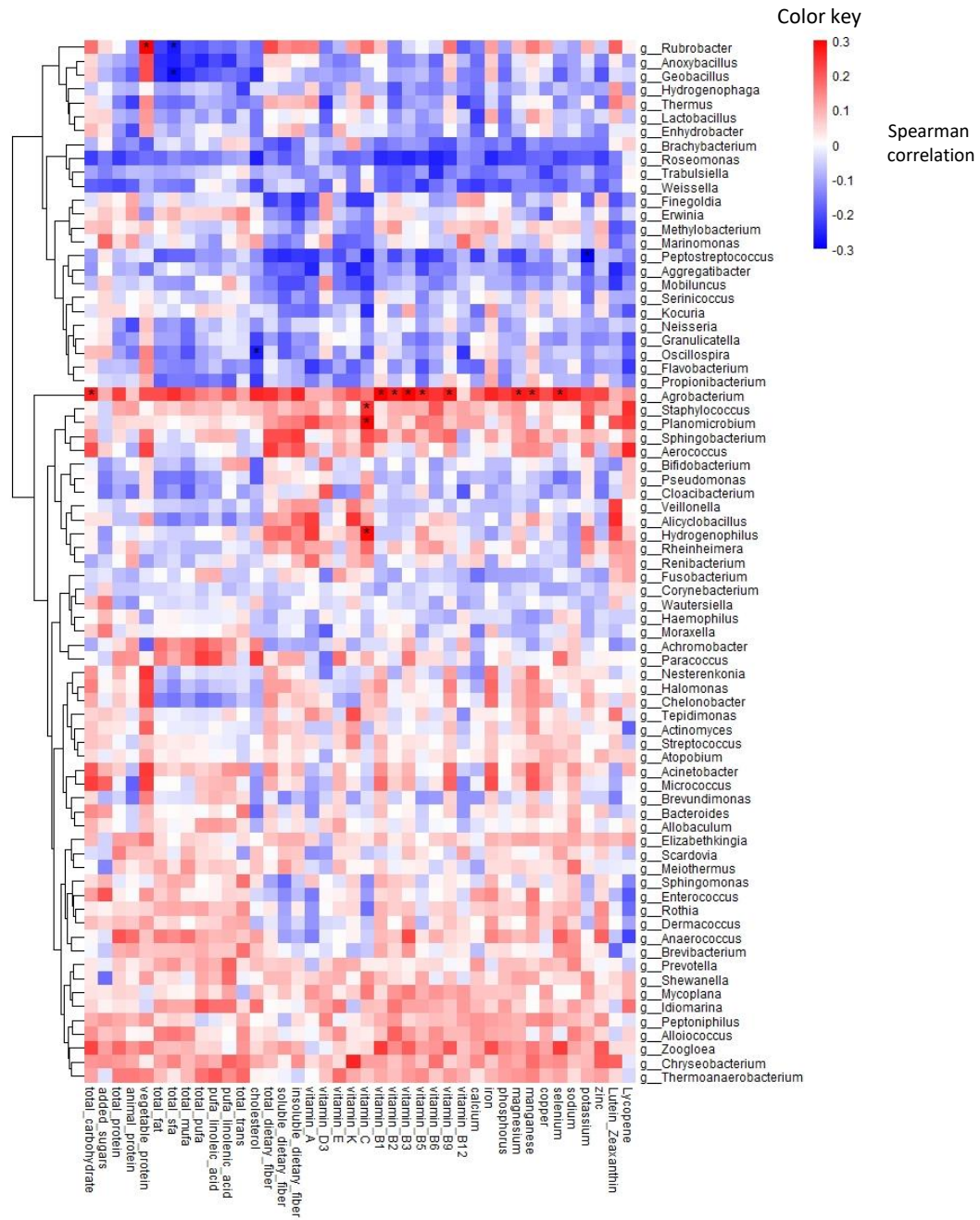

Supplement: Supplementary file 1 [file microorganisms-07-00502-s001.zip › Figure_S4.pdf]

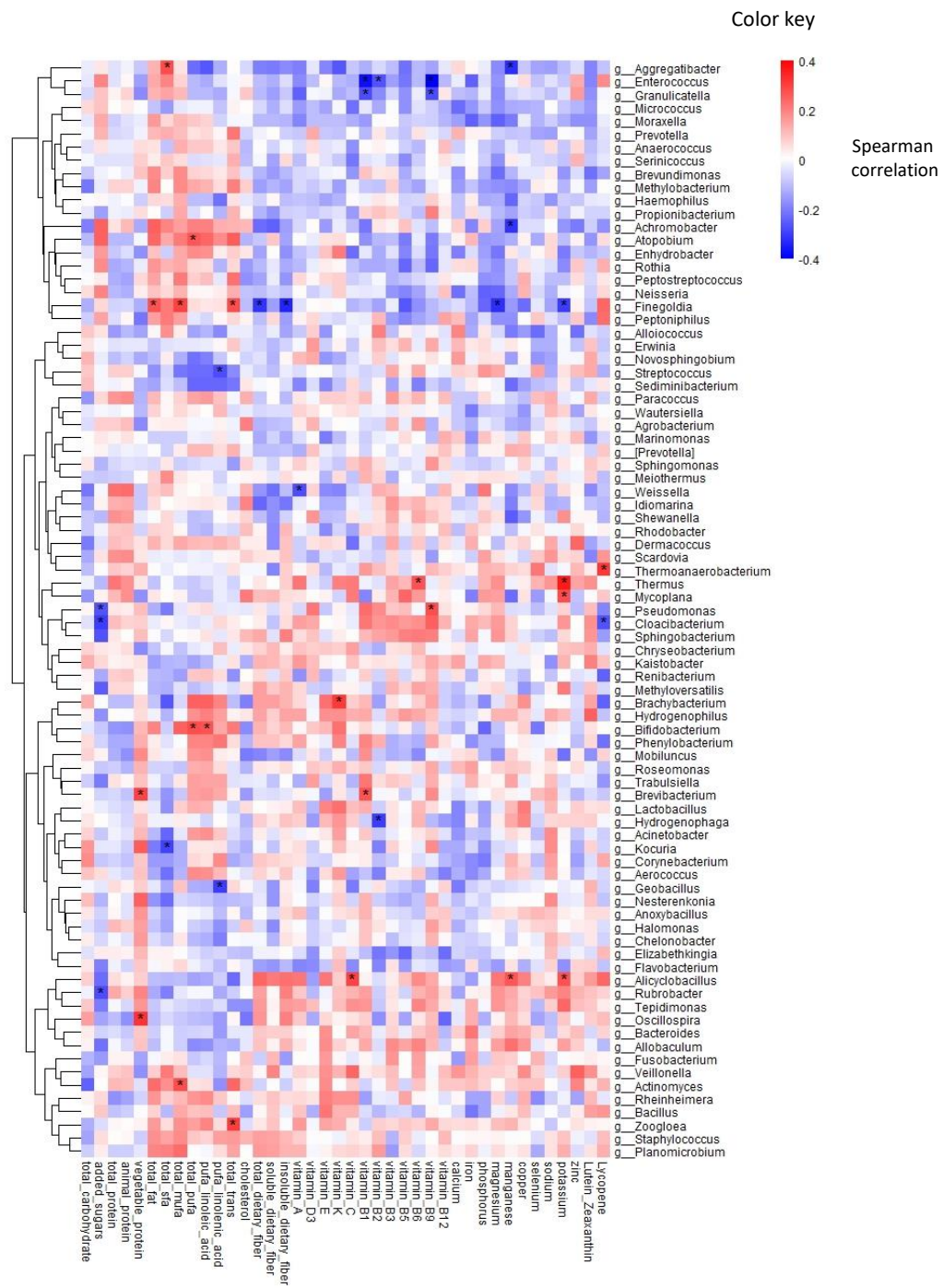

Supplement: Supplementary file 1 [file microorganisms-07-00502-s001.zip › Figure_S5.pdf]

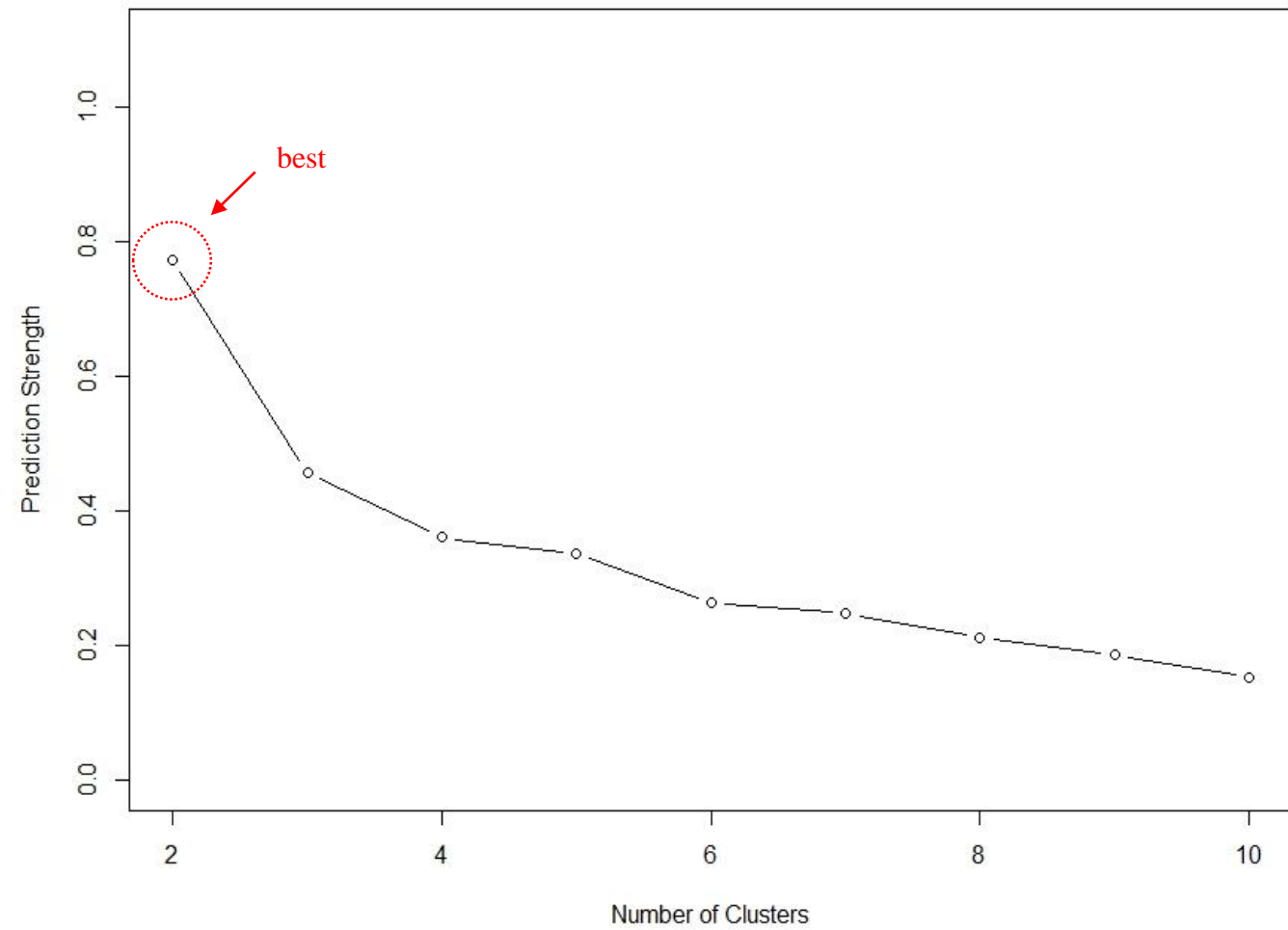

Supplement: Supplementary file 1 [file microorganisms-07-00502-s001.zip › Figure_S6.pdf]

**A.**

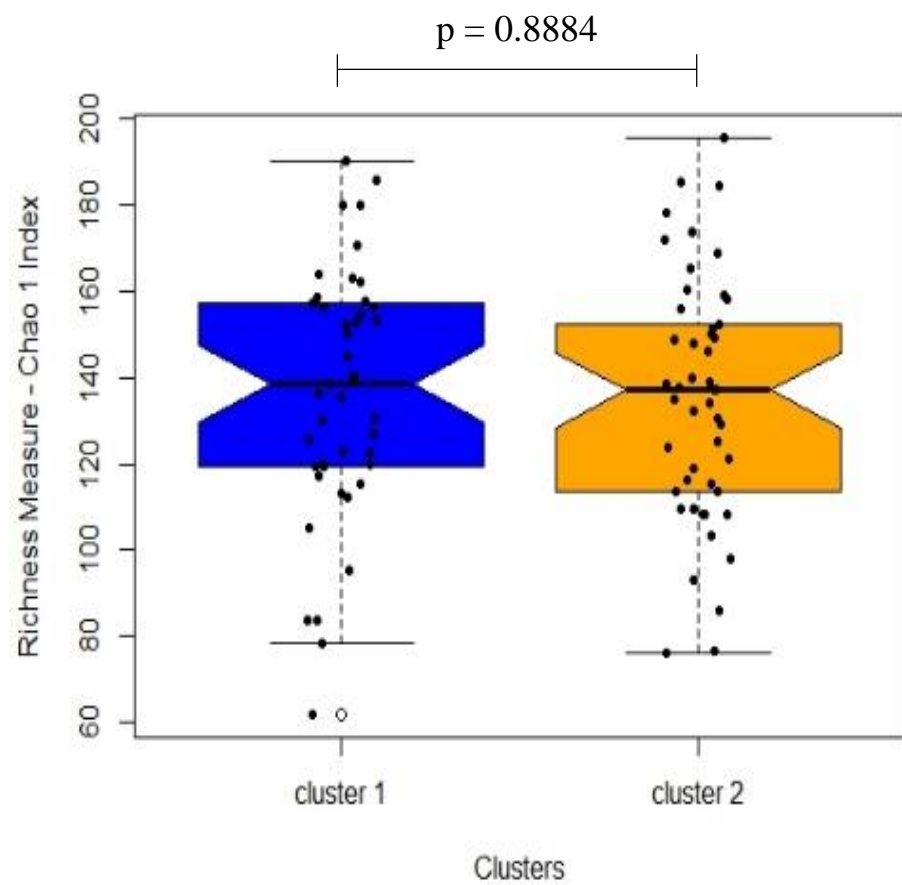

**B.**

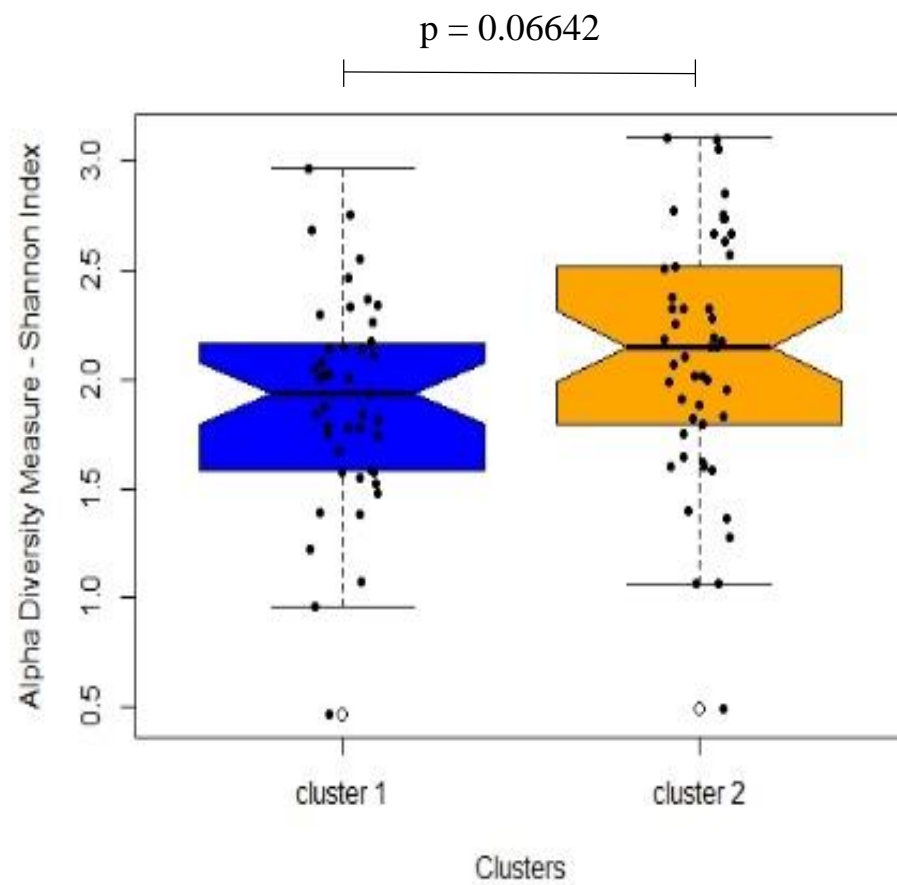

Supplement: Supplementary file 1 [file microorganisms-07-00502-s001.zip › Figure_S7.pdf]
